# An adapted white-coat and warm-heart intervention on nurses’ knowledge, general stigmatizing attitudes, and work avoidance behaviors towards HIV: a quasi-experimental study

**DOI:** 10.1093/joccuh/uiae041

**Published:** 2024-07-22

**Authors:** Ming Yang, Ling Zhang, Ying Jiang, Peng Liu, Wanli Liu, Xiaoxia Cao, Qin Luo, Cangmei Fu, Lianxiang He

**Affiliations:** Teaching and Research Section of Clinical Nursing, Xiangya Hospital of Central South University, Changsha 410008, China; Teaching and Research Section of Clinical Nursing, Xiangya Hospital of Central South University, Changsha 410008, China; Teaching and Research Section of Clinical Nursing, Xiangya Hospital of Central South University, Changsha 410008, China; Teaching and Research Section of Clinical Nursing, Xiangya Hospital of Central South University, Changsha 410008, China; Teaching and Research Section of Clinical Nursing, Xiangya Hospital of Central South University, Changsha 410008, China; Teaching and Research Section of Clinical Nursing, Xiangya Hospital of Central South University, Changsha 410008, China; Teaching and Research Section of Clinical Nursing, Xiangya Hospital of Central South University, Changsha 410008, China; Department of Oncology, the Second Xiangya Hospital of Central South University, Changsha 410012, China; Teaching and Research Section of Clinical Nursing, Xiangya Hospital of Central South University, Changsha 410008, China; Department of Nursing, Xiangya Changde Hospital, Changde 415009, China

**Keywords:** adapted white-coat and warm-heart intervention, POL, HIV, stigma, nurses

## Abstract

**Objectives:**

To determine the effect of an adapted white-coat and warm-heart intervention (AWWI) among nurses.

**Background:**

HIV discrimination among medical staff hinders progress in HIV prevention.

**Methods:**

A total of 779 nurses were randomized into intervention and control groups. The intervention group was provided with AWWI training. The control group did not receive AWWI training. HIV-related knowledge, attitudes, and behaviors of participants were assessed.

**Results:**

Participants in the intervention group had better HIV-related knowledge and less stigmatizing attitudes and work avoidance behavior levels than participants in the control group after the 1-, 3-, and 6-month interventions (*P* < .05). The main effects of group and time factors were highly significant in the intervention group. There were significant interaction effects in group and time factors.

**Conclusions:**

AWWI effectively improved the level of HIV-related knowledge and reduced general stigmatizing attitudes and work avoidance behaviors among nurses based on self-reported data in a tertiary hospital in China during a 6-month period.

## Key points

- **What is already known on this topic:** HIV discrimination among medical staff hinders progress in HIV prevention. The popular opinion leader (POL) intervention model has been confirmed to be an effective behavioral intervention in the prevention and control of HIV. The POL intervention model has been applied in the White-coat and Warm-heart Intervention (WWI) Project to reduce stigma among health care providers in county-level hospitals in China.
- **What this study adds:** The adapted (A)WWI Project was evaluated among nurses at a tertiary hospital in China to test its efficacy in reducing HIV discrimination among nurses. The AWWI improved the level of HIV-related knowledge in nurses and reduced general stigmatizing attitudes and work avoidance behaviors in a 6-month period.
- **How this study might affect research, practice, or policy:** The AWWI Project may deserve further application in multiple tertiary hospitals in China. A multicenter validation may promote the AWWI Project to tertiary and county hospitals worldwide and benefit medical workers caring for people with HIV.

## Introduction

The HIV epidemic is one of the most challenging public health problems worldwide.^1^^,^^2^ Transmission is characterized by the spread from high-risk groups to the general population and is influenced by several factors.^1–3^ Specifically, social stigma is a key factor that contributes to increased HIV transmission.^2^^,^^4^ In the UNAIDS draft guidelines for identifying discrimination related to HIV,^5^ “discrimination related to HIV” is defined as “unfairly differentiated treatment based on established or suspected HIV serology or health status in the same circumstances.” This stigma has resulted in declining hospital care, mandatory testing, excessive precautions, breach of confidentiality, blaming of individuals, and even isolation.^4^^,^^6^^,^^7^ Moreover, the stigma toward people living with HIV (PLH) among health care providers may discourage people from seeking HIV testing, counseling, and access to treatment and care.^7^^,^^8^ Consequently, such stigmatization may further promote the spread of HIV infection in an inconspicuous manner.

HIV-related stigma is associated with a lack of HIV-related knowledge, fear, and misconceptions about the possibility of HIV transmission and prejudice toward risk behaviors.^8–10^ A systematic review concluded that successful HIV-related stigma interventions include peer education, knowledge modules, multimedia stigma reduction training, popular opinion leaders (POLs), and stigma-free space intervention.^11^ However, most approaches to reducing HIV-related stigma worldwide have focused only on HIV knowledge, skill-building, and protecting PLH policies, which can improve short-term knowledge but cannot lead to sufficient behavioral changes.^8–10^ The POL intervention model has been confirmed to be an effective behavioral intervention in the prevention and control of HIV.^12–17^

POLs are leaders in this field and are respected, trusted, and influential among peers. POLs have a crucial role in disseminating training information to peers in their work or life. Using their words and actions, POLs administer continuous intervention to their peers and gradually influence peer behavior and change their habits. Based on the framework of innovation theory diffusion,^15^ the POL intervention model was applied to the White-coat and Warm-heart Intervention (WWI) Project to reduce stigma among health care providers in county-level hospitals in China.^14^^,^^16^ The WWI Project is a specific tool comprised of behavioral- and structural-level components.^14^^,^^16^ Infusion theory emphasizes that new behavioral trends are most efficiently established when a critical mass of POLs adopt and endorse the new trend in the community for the behavioral-level components. Of the service providers within each unit of the hospitals, 15% were nominated as POLs by the administrator to participate in the WWI training program. These POLs were requested to train peers, convey stigma reduction messages, and demonstrate universal precautions to their peers in their daily work and life to raise awareness of compliance with universal precautions and alter risk behavior of PLH. WWI emphasizes the importance of compliance with universal precautions when caring for PLH and facilitation of caring for infectious patients with HIV among nurses using personal protective equipment for the structural-level components. Studies have shown that this WWI project is effective in reducing HIV/AIDS stigma^14^^,^^16^.

However, the WWI Project has not been applied among nurses in the tertiary hospitals of China. The clinical situation in tertiary hospitals differs from county hospitals with respect to social context, service scope, education level of medical staff, and hospital support system. Therefore, the WWI Project has been revised and improved to become the adapted (A)WWI Project to provide better suitability for use in tertiary hospitals and nurses in China. The efficacy of the AWWI Project on the knowledge, stigmatizing attitudes, and behaviors related to HIV care in a tertiary hospital among nurses was analyzed in this study.

## Methods

### Contextual background

The WWI Project has been implemented among physicians, nurses, and technicians in 40 county hospitals in China, and encouraging outcomes have been demonstrated in previous studies.^14^^,^^16^

However, tertiary hospitals differ from county hospitals with respect to social context, service scope, education level of medical staff, and hospital support system. Moreover, the duties of nurses are different from those of physicians and laboratory technicians. Therefore, an AWWI has been revised and improved from the WWI to provide better suitability for use in tertiary hospitals and nurses. Based on the Map of the Adaptation Process (MAP) developed by the US Centers for Disease Control and Prevention,^16^ adaptation is defined as the process of modifying key characteristics of an intervention, recommended activities, and delivery methods without competing with or contradicting the core elements and internal logic of the intervention. The core elements of the WWI were retained in the AWWI Project, including the personal occupational safety of health care providers, the importance of regarding all patients as infected patients, and compliance with universal precaution during the process of caring for patients. Additionally, the AWWI Project added or removed content, such as modifications to existing role-playing cases, games, videos, and cultural modifications based on MAP assessment.

Hunan, an area in south-central China with 70 million residents, has a high HIV prevalence. By October 31, 2022 there were 53 030 living HIV/AIDS cases in Hunan province. The Xiangya Hospital is a large general hospital under the National Ministry of Education and Ministry of Health in China. The Xiangya Hospital ranks first in Hunan province based on its comprehensive strengths. The hospital has 3500 beds with 90 units and a medical staff of more than 6000 individuals, including approximately 2650 nurses. Clinical front-line nurses are likely to encounter patients with HIV or unknown HIV status when the latter are admitted to the hospital. Therefore, the AWWI Project was conducted in the hospital.

### Ethical approval

The protocol of this study was approved by the Ethics Committee of Xiangya Hospital (No. 201702019). All methods were carried out in accordance with relevant guidelines and regulations. The participants signed written informed consent before the study commenced.

### Study design and settings

A quasi-experimental design with intervention and control nursing units was used to measure the effect of stigma-reduction intervention on nurses. The intervention was implemented in 40 nursing units in the hospital from March 2019 to February 2020. Each participant signed a written informed consent form.

### Study participants

**Figure 1:**
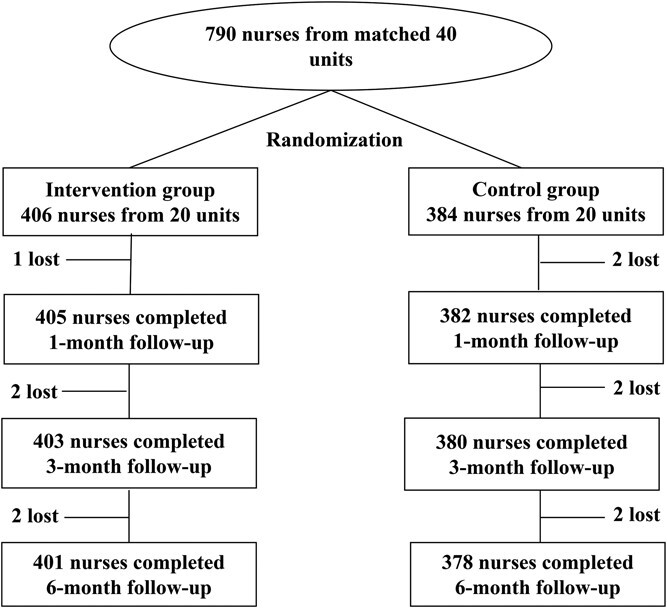
Flow chart of study participants.

The hospital units were matched pairwise based on the number of beds, service scope, size of the nursing staff, nursing services offered, potential access to HIV-infected patients, and number of patients with HIV infections (except in the emergency room, operating room, and ICU units). Twenty pairs of matched nursing units (40 units) were selected from 68 hospital departments by purposive sampling. The 40 units were randomized to the intervention or control group, and each group was comprised of 10 surgical units, 8 internal medicine units, and 2 obstetrics and gynecology units. Staggered floors were considered between the intervention and control nursing units to avoid potential contamination. The recruited nurses had >1 year of first-line clinical experience in the hospital. Nurses who could not participate in the entire research process were excluded. Nurses who were unable to participate fully in the study were excluded, including intern nurses and those on maternity leave or business trips. Researchers explained the purpose of the study, procedures, voluntary participation, and potential risks and benefits to the participants. Written informed consent was obtained from each participant. The 40 units yielded 790 study participants, 779 of whom completed the 6-month follow-up evaluation. The study included 401 and 378 participants in the intervention and control groups, respectively (Figure 1).

### The AWWI

The adaptation steps of the AWWI Project involve assessment, expert workshop, training trainers, preparation, and pilot, which have been previously described.^14^^,^^16^^,^^18^^,^^19^

The first step was assessment, which involved evaluation of the target population requirement, availability of the potential tertiary hospital resources (eg, staff, space, and fiscal), and the experience of implementing the WWI intervention as well as specific intervention skills. Thirty clinical first-line nurses underwent in-depth interviews and 7 nurse managers and directors of related management departments participated in the focus group, which focused on standard prevention, occupational exposure, and factors influencing discrimination against HIV-infected individuals. Valuable information was integrated into the WWI to produce the AWWI revision 1.

The second step was the expert workshop. Eleven experts with multidisciplinary experience were invited to discuss the AWWI revision 1. The experts included the supervisor of the author team (Dr Li) and one important member who oversaw the original WWI program, and 8 clinical nursing experts from the hospital infection center, operating room, and emergency, vascular surgery, dermatology, obstetric, nursing, and respiratory departments. The 8 nursing experts comprised the team of facilitators. Feedback was integrated into the AWWI revision 1 to create AWWI revision 2.

The third step involved the training of facilitators. The 8 facilitators of AWWI recruited at the second step were divided into 4 groups. Each group received 1 session of AWWI demonstration teaching fully in accordance with Revision 2, whereas the supervisor of the author team, Dr Li, and one important member who were the performers of the original WWI program were invited to conduct on-site training and supervision. After each session, the facilitators received suggestions from the principal investigator and supervisors on training skills, including allocation of appropriate time for each part of each session to control the training process, strategies to inspire trainees to participate actively throughout the session, focusing on the summary in each part, and a review by the end of each session. Feedback from each trainee on the training content, activities, and methods, among other components, was also obtained to improve AWWI revision 2 and create revision 3.

The fourth step involved preparation for implementing the intervention, included the training site, materials, and recruitment of POLs.

The fifth step was the pilot study. Ten POLs were selected using objective sampling to ensure that the pilot participants represented the intervention target population. The 8 facilitators (recruited in the third step) conducted 4 sessions for the 10 POLs, fully adhering to revision 3, for 2 days. Two facilitators were responsible for 1 session (90 minutes in length). After each session, feedback on the training content, activities, and methods, among other parameters, was collected from the facilitators and POLs. The feedback was analyzed to identify the need for removal, addition, or modification of materials in revision 3 to enhance the relevance and efficacy for the target population. Consequently, the final revision of the AWWI was generated.

The specific adapted contents of the WWI are presented in Table S1. The final AWWI included the following 4 sessions: (1) complying with standard precaution procedures and ensuring nursing safety; (2) fighting against stigma and improving the patient-provider relationship; (3) taking actions and making efforts to care for patients; and (4) overcoming difficulties and building a better medical environment.

### AWWI intervention

In this study the AWWI Project was used as a specific intervention.

Based on Rogers' diffusion of innovation theory,^15^ a POL intervention model was developed in this study. POLs were defined as service providers who are trustworthy, respected, and influential among colleagues, and whose suggestions would be seriously considered by their co-workers. To identify POLs, recruitment notifications were issued to each unit of the intervention group. The nurse manager recommended 15% of suitable POL nurses with different professional titles (junior, senior, and chief nurses) in each unit with informed consent. From these voluntary nurses, we recruited approximately 2-4 POLs from each unit (total = 62 POLs).

A total of 406 participants, including the 62 POLs in the intervention group, received the AWWI training, which included an initial training of 4 weekly sessions over a 1-month period. Each session lasted approximately 90 minutes and was conducted in a classroom located away from the unit. The sessions featured issue-oriented games, group discussions, role playing, and video mode instruction. The facilitators focused on mentoring and encouraged the POLs to think, communicate, and share.

The 62 POLs in the intervention group were instructed to disseminate the message to their colleagues and demonstrate skills for their peers in the work site after each session. POLs established goals for engaging in informal conversations with colleagues after weekly sessions, and the conversational outcomes were discussed at the next session. The training information was communicated to colleagues in a timely fashion and related skills of standard prevention were demonstrated in daily work. Communication skills were emphasized to help the POLs effectively deliver the intervention messages to their co-workers. For example, the POLs were advised to use the project logo to initiate conversation to advocate for the project, and they were encouraged to disseminate intervention information from the point of view of nurse compliance with standard precautions and treat all patients equally. This was related to their daily work and required continuous information dissemination and behavioral intervention of peers.

At the 1-, 3-, and 6-month time points after the initial training, POLs in the intervention group were convened to participate in a separate, 90-minute reunion session. At each reunion, POLs were encouraged to share their experience, difficulties, and countermeasures of disseminating prevention standard and anti-discrimination behaviors among colleagues. In addition, 1 individual with an HIV infection and a nurse manager in the HIV unit from another hospital treating infectious diseases were invited to participate in 3 group reunions and share their experiences in the reunion face-to-face. The POLs were expected to enhance the empathy and understanding of PLH, correct misconceptions of HIV transmission, and eliminate their fear of caring for HIV patients in the reunion session.

In the control group training on the AWWI was not provided and only follow-up visits and assessments were performed.

### Assessments and follow-up evaluations

At baseline and at the 1-, 3-, and 6-month assessments, each participant from the intervention and control groups had follow-up evaluations and completed a self-administered paper-and-pencil questionnaire in a private room with a trained interviewer available to answer questions. The survey took an average of 15 minutes to complete. The follow-up rate was >98% (779/790) in the 2 groups.

The following 3 scales, which were developed by Li et al^14^^,^^16^ of the University of California at Los Angeles, were used in this study. The WWI program research team first applied the scales in the HIV stigma study of the medical staff in China in 2013, with Chinese and English versions attached.^14^^,^^16^

Knowledge of HIV was determined by Professor Li.^14^^,^^16^^,^^18^^,^^19^ The retest reliability (Cronbach α = .80) of the scale was retested.^18^^,^^19^ Knowledge of HIV was measured using 10 questions in the version used by Professor Li^14^^,^^16^^,^^18^^,^^19^ but included 12 questions in the AWWI Project. These questions have been used, together or separately, in many studies involving HIV to measure HIV-related knowledge. For each item, the responses were coded as 1 (correct answer) or 0 (incorrect answer or unknown). The scale for knowledge of HIV/AIDS was constructed as a sum of all 12 items. A higher score indicated a higher level of HIV-related knowledge. Regarding the reliability of the scale of knowledge of HIV/AIDS, prior to and during the design of the program, we consulted with Professor Li, the leader of the original WWI program, who replied that knowledge-based questionnaires do not generally require reliability testing.

A general prejudicial attitude scale was adapted by Li et al^14^^,^^16^ from the HIV/AIDS-related Stigma and Discrimination Indicators Development Workshop Report (Cronbach α = .73), with high scores in the adapted version implying high levels of prejudicial attitude. The general prejudicial attitude variable was measured based on 8 items that were scored from 1 (strongly agree) to 5 (strongly disagree). Example items included “people who got HIV/AIDS through sex or drug use got what they deserved” and “HIV is a punishment for bad behavior.” The directions of some items were reversed so that the higher score indicated a higher degree of general prejudicial attitude. The scale was tested in our pilot study to ensure culture relevancy, and the retest reliability of the scale was .81, which indicated good stability. The scale has been validated by repeated application in subsequent studies with good reliability (Cronbach α = .75)^18^^,^^19^.

An avoidance behavior scale was modified from Herek by Li et al^14^^,^^16^^,^^18^^,^^19^ and the WWI Project. The avoidance behavior scale was based on 8 items; the responses to each statement ranged from 1 (strongly agree) to 5 (strongly disagree). Eight hypothetical situations were listed to assess the level of avoidance behavior toward PLH when participants performed their daily nursing work. Example statements in this scale included, “If HIV-positive patients visit the hospital, you are willing to provide all service needed” and “If you worked with HIV-positive patients, you would provide the same quality of care to them that you provide to other patients.” The reliability (Cronbach α = .84) and retest reliability (Cronbach α = .83) of the scale were retested.^18^^,^^19^ The assessment tool used in this study was shown to have good credibility, which not only ensured the reliability of our results, but also demonstrated the extensibility of AWWI training.

We also included variables on respondents’ demographic information, such as age, gender, educational experience, departments, work experience, and experience of exposure to or caring for an HIV-infected person, whether they have treated or been exposed to PLH, whether they have received HIV-related training (yes or no), and available access to receive HIV infection training.

### Statistical analysis

Data were entered and analyzed using SPSS (version 22). We used *t* tests and a c^2^ test or the Wilcoxon signed-rank test to analyze categorical and continuous variables for baseline characteristics of nurses from the intervention and control groups. Repeated measures analysis of variance (ANOVA) was used to examine the overall mean HIV stigma scores, including HIV knowledge, attitudes, and behavior, for the intervention and control groups at baseline and at the 1-, 3-, and 6-month follow-up assessments. A contour diagram was drawn to describe the changing trend of the total mean HIV knowledge, attitude, and behavior scores for both groups at the 4 time points. Repeated measures ANOVA was used to compare and analyze the total mean HIV knowledge, attitude, and behavior scores in the intervention and control groups at different time points. The Mauchly test of sphericity was applied to the data. The observed values did not satisfy the spherical symmetry of the covariance matrix. A *P* value <.05 was considered a statistically significant difference.

## Results

Nearly all participants were females and had a median age of 31 years (range, 21-55 years). The average work experience was approximately 9 years. Greater than 80% of participants reported prior experience caring for PLH. Participants in the intervention and control groups were similar with respect to age, gender, education level, professional title, years of nursing service, and nursing units at baseline. None of the characteristics at baseline showed statistically significant differences, as shown in Table 1.

**Table 1:** Demographic and background characteristics by group at baseline.

**Characteristic**	**Control (*n* = 378)** **No. (%) or mean ± SD **	**Intervention (*n* = 401)** **No. (%) or mean ± SD **	***P***
**Gender: female**	378 (100)	399 (99.5)	.500^a^
**Age, y**			
**≤30**	248 (65.6)	256 (63.8)	.266^a^
**31-40**	93 (24.6)	91 (22.7)	
**≥41**	37 (9.8)	54 (13.5)	
**Years of working**			
**≤10**	284 (75.1)	293 (73.1)	.459^a^
**11-20**	60 (15.9)	61 (15.2)	
**≥21**	34 (9.0)	47 (11.7)	
**Education**			
**Secondary**	0 (0.0)	2 (0.5)	.403^b^
**Associate’s degree**	83 (22.0)	97 (24.2)	
**Bachelor’s degree**	274 (72.4)	280 (69.8)	
**Master’s degree**	21 (5.6)	22 (5.5)	
**Professional role**			
**Nurse**	39 (10.3)	44 (11.0)	.703^b^
**Senior nurse**	233 (61.6)	235 (58.6)	
**Supervisor nurse**	99 (26.2)	118 (29.4)	
**Co-chief superintendent nurse**	6 (1.6)	4 (1.0)	
**Chief superintendent nurse**	1 (0.3)	0 (0.0)	
**Department**			
**Surgery**	206 (54.5)	215 (53.6)	.918^a^
**Internal medicine**	142 (37.6)	153 (80.5)	
**Obstetrics-gynecology**	30 (7.9)	30 (7.5)	
**Previous contact with HIV-infected individuals**			
**Yes**	311 (82.3)	323 (80.5)	.581^a^
**No**	67 (17.7)	78 (19.5)	
**Primary measures at baseline**			
**Knowledge of HIV/AIDS**	8.80 ± 2.05	9.02 ± 1.65	.098^b^
**General prejudiced attitude**	22.29 ± 5.98	22.53 ± 5.20	.551^b^
**Avoidance behavior**	20.19 ± 4.53	20.56 ± 4.34	.253^b^

a c^2^ or Fisher exact test.

b Wilcoxon signed-rank test or 2-group *t* test.

### Time trend

**Figure 2:**
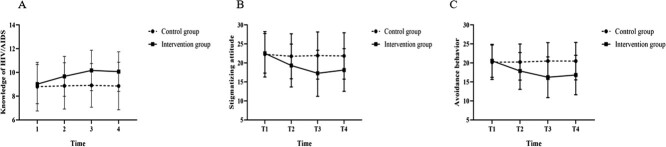
Plots of means (±SD) over time for knowledge, stigmatizing attitude, and avoidance behavior. A, Knowledge of HIV/AIDS; B, stigmatizing attitude; and C, avoidance behavior. T1, time point before training; T2, time point after the first month of training; T3, time point after the third month of training; T4, time point after the sixth month of training.

The total score of HIV-related knowledge showed an upward trend after the intervention, and the overall level of HIV-related knowledge increased. The knowledge score was highest 3 months after the intervention (Table 2). The stigmatizing attitude and work avoidance behavior characteristics in the intervention group were significantly reduced. The overall scores of the general prejudicial attitude scale and work avoidance behavior decreased, and the total scores of the general prejudicial attitude scale and work avoidance behavior were lowest in the 3 months after the intervention compared with before the intervention (Table 2). The scores for HIV-related knowledge, general stigmatizing attitude, and work avoidance behavior remained steady at all time points in the control group and there were no statistical differences in the trend of changes at each time point (Figure 2).

**Table 2:** Knowledge of HIV, general prejudicial attitude scale, and avoidance behavior scale scores for 4 time points (mean ± SD).^a^

	**T1**	**T2**	**T3**	**T4**
**Intervention (*n* = 401), mean ± SD**				
**Knowledge of HIV/AIDS**	9.02 ± 1.65	9.67 ± 1.68	10.17 ± 1.70	10.07 ± 1.67
**General prejudicial attitude scale**	22.53 ± 5.20	19.32 ± 5.64	17.28 ± 6.07	18.14 ± 5.61
**Avoidance behavior**	20.56 ± 4.34	17.89 ± 4.84	16.23 ± 5.36	16.83 ± 5.19
**Control (*n* = 378), mean ± SD**				
**Knowledge of HIV/AIDS**	8.80 ± 2.05	8.87 ± 1.95	8.91 ± 1.84	8.86 ± 2.00
**General prejudicial attitude scale**	22.29 ± 5.98	21.76 ± 5.92	21.94 ± 6.21	21.84 ± 6.10
**Avoidance behavior**	20.19 ± 4.53	20.24 ± 4.74	20.51 ± 4.86	20.48 ± 4.93

a T1, time point before the training; T2, time point after the first month of training; T3, time point after the third month of training; T4, time point after the sixth month of training.

### Intervention effects

The degrees of freedom were corrected by Epsilon, and the results of Greenhouse-Geisser correction were used. The results of repeated measures ANOVA of the HIV knowledge, attitude, and behavior total scores are shown in Table 3. The interactive contour diagram of groups and time are shown in Figure 2. The main effect of group factors was significant for HIV/AIDS knowledge (*F* = 81.446, *P* < .001). The main effect of time factors was significant (*F* = 31.455, *P* < .001). The interaction of groups and time factors was significant (*F* = 22.460, *P* < .001). All of the above differences were highly statistically significant. The main effect of group factors was significant for stigmatizing attitude (*F* = 61.341, *P* < .001). The primary effect of time factors was significant (*F* = 74.909, *P* < .001). The interaction of group and time factors was significant (*F* = 54.430, *P* < .001). All these differences were highly statistically significant. The main effect of group factors was significant for avoidance behavior (*F* = 82.976, *P* < .001). The main effect of time factors was significant (*F* = 48.077, *P* < .001). The interaction of group and time factors was significant (*F* = 65.495, *P* < .001). Each of these differences was considered highly statistically significant. The changes in the trend of HIV-related knowledge, general prejudicial attitude scale, and work avoidance behaviors in the 2 groups were statistically different at different time points (Figure 2).

**Table 3:** Results from ANOVA for repeated measurements.^a^

**Outcome measures**	**T1**	T2	T3	T4	**Greenhouse-Geisser**	
	**Mean ± SD**	**Mean ± SD**	**Mean ± SD**	**Mean ± SD**	***F***	***P***
**Knowledge of HIV/AIDS**	9.02 ± 1.65	9.67 ± 1.68	10.17 ± 1.70	10.07 ± 1.67		
**Group factors**					81.446	.000
**Time factors**					31.455	.000
**Interaction**					22.460	.000
**Stigmatizing attitude**	22.53 ± 5.20	19.32 ± 5.64	17.28 ± 6.07	18.14 ± 5.61		
**Group factors**					61.341	.000
**Time factors**					74.909	.000
**Interaction**					54.430	.000
**Avoidance behavior**	20.56 ± 4.34	17.89 ± 4.84	16.23 ± 5.36	16.83 ± 5.19		
**Group factors**					82.976	.000
**Time factors**					48.077	.000
**Interaction**					65.495	.000

Abbreviation: ANOVA, analysis of variance.

a T1, time point before the training; T2, time point after the first month of training; T3, time point after the third month of training; T4, time point after the sixth month of training.

## Discussion

The results in this study showed that AWWI intervention not only increased knowledge of HIV-related information and reduced nurses’ stigma towards HIV but also improved the avoidance behavior of nurses over a 6-month period. The results of the study are encouraging and warrant further replication of this intervention model.

Our findings were consistent with previous studies,^10^^,^^11^^,^^20^^,^^21^ which have indicated that some interventions can improve the knowledge of HIV-related information and stigmatizing attitudes among nurses. In contrast to related studies, our study further changed the stigmatizing behaviors effectively. This specific effect exerted by the AWWI intervention may be associated with the following factors. First, the AWWI sustained the core component of the WWI and was dedicated to the occupational self-protection and safety of nurses.^14^^,^^16^ A fear of HIV transmission was identified as a primary driver of stigma toward PLH in the health care setting. All participants in the AWWI Project were required to fully comply with the standard precautions while performing their routine duties, and the training sessions enhanced their knowledge about HIV so that their stigma toward PLH was significantly reduced. Second, the reasonable arrangement of POLs had an important role in the training process. POLs are considered respectable, reliable, and influential by their colleagues.^12–14^ When POLs were selected for training, they were full of a sense of pride, responsibility, and mission, and had positive attitudes and a strong desire to acquire knowledge and skills during training. The POLs were from various levels in the same unit, had more common language with peers, and were familiar with peer strengths and weaknesses. Therefore, their words and deeds were appealing and influential among the peers so that they spread new ideas, knowledge, and techniques easily.^11–14^ Third, the sessions were reasonably arranged. POLs were selected based on their various professional titles in different units. Convenient training times were arranged to ensure 100% participation of POLs and the participants in the intervention group for the 4 training sessions. The tables and chairs were arranged in the shape of a U for participants to communicate face-to-face and facilitate their participation and interaction with each other in each training session. Fourth, situational case presentation and sharing in the training and reunion session helped inspire real feelings and empathy among the POLs and the intervention group. The AWWI training incorporated games, group discussion, role-play, and real-life cases from the hospital to inspire full participation of the participants in the intervention group.^11^ Moreover, it was convenient for POLs to comprehend and find problems and disseminate interventional content according to their department's status. Therefore, POLs received great approval from peers in clinical work and internalized the same.

Davtyan et al^22^ found that the intervention effectiveness was reduced at the third month among health care providers. The DriSti intervention studies demonstrated significant reductions of misconceptions and worrying about acquiring HIV at work among health care professionals at the 6-month follow-up assessment.^23^^,^^24^ It is worth noting that the intervention effects of AWWI were durable for 6 months. Three reunion sessions were conducted after 1, 3, and 6 months to ensure the sustainability of message diffusion. According to forgetting curve theory, forgetting starts immediately after learning the action. Specifically, 66.3% is forgotten after 1 day, 74.6% after 6 days, and approximately 79% after 31 days.^25^ The reunion sessions are an important platform of brainstorming for the POLs to share their experiences and problems during the period of message dissemination and skill demonstration, which facilitates an understanding of learning in POLs and enhances their confidence in continuing the dissemination of information and relative behaviors among peers.^26^ Especially, POLs may alter peer awareness through repeated diffusion.^14^ Thus, reunion sessions are repetitive memory-enhancing processes that are instrumental in sustaining beneficial intervention effects. Training skills have also been confirmed to be an important aspect for promising intervention outcomes. Trainers affectionately refer to POLs as “messengers of love” and inspire trainees to always remember their mission and spread love.^11^^,^^12^ The trainers paid attention to the participation of each POL nurse. For inactive or silent POLs, the trainer provided appropriate encouragement and opportunities to promote positive participation and free expression. Finally, nurses were also provided with adequate protective equipment to protect them against occupational exposure and to reduce their psychological burden.^19^ The overall study protocol was carried out under repeated, strict discussion and supervision of the supervisor of the author team, Professor Li, who initiated the WWI Project,^14^^,^^16^ and a senior statistical expert, who considered the follow-up period to be reasonable.

This analysis had several limitations. First, data were only collected from nurses in 1 tertiary hospital. In future studies, we hope to extend the study to nurses from multiple tertiary hospitals. Second, outcome evaluation depended only on self-reported data, and social expectation bias could be an issue. Finally, no definitive information was available on whether the quality of care for PLH improved after the intervention. Despite these limitations we showed that the AWWI was feasible and effective in a tertiary hospital in China. As the demand for HIV care increases, nurses are under great pressure to deliver positive services. The AWWI may have the potential to help the nurses in other tertiary hospitals to reduce their general stigmatizing attitudes toward PLH.

## Conclusion

Based on the self-reported data of the nurses, the AWWI Project effectively improved HIV-related knowledge, reduced general stigmatizing attitudes and work avoidance behaviors, and helped induce a 6-month positive effect among the nurses in a tertiary hospital in China. Although lacking information from patient-reported data and about improvements in quality of care for PLH, the efficacy of the AWWI Project deserves further application and validation in multiple tertiary hospitals in China.

## Supplementary Material

Supplemental_Table_1_uiae041

## Data Availability

The datasets generated during and/or analyzed during the current study are not publicly available, but are available from the corresponding author on reasonable request.
