# Supplementary material for: An adapted white-coat and warm-heart intervention on nurses’ knowledge, general stigmatizing attitudes, and work avoidance behaviors towards HIV: a quasi-experimental study
Source: J Occup Health. 2024 Jul 22;66(1):uiae041. doi: 10.1093/joccuh/uiae041 (PMC11360590; doi:10.1093/joccuh/uiae041)
Supplement: Supplemental_Table_1_uiae041 [file supplemental_table_1_uiae041.docx]

**Supplemental Table 1. Main adapted content of WWI**

| **Session** | **Main adapted content** | **Reason for adaptation** |
| --- | --- | --- |
| 1. Compliance with standard precaution procedures and ensuring nursing safety | ① Replacing the video scene: "The finger of a laboratory technician was pricked by the glass and stained the patient blood" and "the patient's blood splashed into the dentist's eyes" were replaced with "the secondary dealing with medical wastes led to occupational exposure," and "the nurse without gloves draws blood for hematological evaluation of patients."  ② Changed the game "five patients with different identities visit a doctor" to "five patients with different identities went to the nurse station for blood drawing"  ③ "occupational exposure of nurses in the secondary separation of clinical waste" instead of "nurse re-set the needle cap after the puncture" | ①② It is not suitable for the working environment of nurses  ③ Original case did not meet the updated guideline for nurses in tertiary hospitals |
| 1. Fighting against stigma and improving the patient-provider relationship | Add the real cases obtained during the interview  (① A patient with AIDS, who was unfit for transfer, was transferred to another hospital, and resultantly, succumbed to death soon; ② The difference between the pre-and post-care operations created and increased the gap) | Improve the understanding of the dangers of discrimination against patients with AIDS among nurses |
| 1. Taking actions and making efforts to care for patients | ①Changed the role-playing case: "Doctors refuse to treat patients suspected with AIDS" was replaced with "When a junior midwife received an HIV-positive woman who was about to give birth, she panicked and was unsure of what to do. The POL nurse immediately gave her a live demonstration. The nurse calmed down and successfully assisted the POL nurse in the delivery"  ② Adding the case discussion "Two medical workers who were talking in whispers at a nurse's station about an HIV-infected middle school boy were overheard by the boy's mother, who was angry and warned the two nurses about the consequences" and "When the nurse spoke to the patient, she inadvertently disclosed the patient's condition, which made the patient depressed and suicidal" | ① The original case focused on doctors rather than nurses  ② Emphasizing that medical staff do not discuss the conditions of patients in public and highlighting the importance of protecting patient privacy |
| 1. Overcoming difficulties and building up a better medical environment | ① Adopting a popular video on the blog and WeChat, in which a blind man sits on the street begging with a small cardboard sign that reads: "I am blind, please help me." Although many people pass by, no one gives alms. As a beautiful woman passes by, she changed the words on the cardboard into: "Today is a nice day, but I can't see it." After that, passers-by stopped and gave alms generously."  ② The game of "actions speak louder than words" was replaced by "Blind men touching an elephant" | ① Emphasizing the importance of language communication skills  ② The POLs were encouraged to disseminate useful information through words, to serve as advocates and role models for action and to influence others through words and deeds |
